# Exploring Influences on Theory of Mind Impairment in Opioid Dependent Patients

**DOI:** 10.3389/fpsyt.2021.721690

**Published:** 2021-11-23

**Authors:** Katharina Eidenmueller, Franz Grimm, Derik Hermann, Ulrich Frischknecht, Christiane Montag, Isabel Dziobek, Falk Kiefer, Nina Kim Bekier

**Affiliations:** ^1^Department of Addictive Behaviors and Addiction Medicine, Central Institute of Mental Health, Mannheim, Germany; ^2^Therapieverbund Ludwigsmühle gGmbH, Landau, Germany; ^3^German Institute of Addiction and Prevention Research, Catholic University of Applied Sciences Nordrhein-Westfalen, Koeln, Germany; ^4^Department of Psychiatry and Neurosciences, Charité University Medicine, Berlin, Germany; ^5^Berlin School of Mind and Brain, Institute of Psychology, Humboldt-Universität Zu Berlin, Berlin, Germany

**Keywords:** theory of mind, opioid dependence, opioid addiction, opioid maintenance treatment, executive functions, childhood maltreatment

## Abstract

Theory of mind (ToM) is an aspect of social cognition impaired in different addictive disorders, including opioid addiction. This study aimed at replicating ToM deficits in opioid dependent patients undergoing opioid maintenance treatment (OMT) and exploring the influence of substance use related variables, executive functions and childhood maltreatment on ToM in opioid dependent patients. 66 opioid dependent patients were tested using the Movie for Assessment of Social Cognition (MASC) and compared with the data of healthy controls. Furthermore, the opioid dependent patients underwent testing for executive functions and filled in the Childhood Trauma Questionnaire (CTQ). Performance on the MASC was significantly poorer in the opioid dependence group than in the control group, even when recent additional drug use and psychiatric comorbidities were controlled for. No correlations were found between ToM and substance use related factors. Aspects of ToM performance in opioid dependent patients correlated significantly with different EF domains. ToM correlated significantly with the CTQ scales for physical maltreatment. The results confirm impaired ToM in opioid dependent patients and highlight executive functions and childhood maltreatment as influential factors. The lack of associations between ToM and substance use related variables and the association with childhood maltreatment suggest that ToM impairments might be a risk factor predating substance abuse.

## Introduction

Theory of Mind (ToM) describes the attribution of mental states to oneself and others. It includes the ability to make inferences about other people's thoughts, emotions, knowledge, expectations, desires, ideas and intentions. These social-cognitive processes play a huge role in human social interactions (1). Impairments in ToM abilities have been shown in patients suffering from schizophrenia (2), bipolar disorder (3), autism spectrum conditions (4, 5) and Huntington's disease (6). So far, studies examining the relationship between impaired ToM and other outcome variables have mostly been conducted in the field of schizophrenia research. In schizophrenic patients, a limited ToM capability was shown to be the best predictor for poor social competence (7). ToM impairments have been associated with limited insight into illness (2) and poor quality of life (8) in schizophrenic patients. As demonstrated by these examples, ToM is a construct that may have an impact on health factors beyond social reciprocity and is therefore worth studying in other illnesses that affect the brain, e.g., psychiatric illnesses, other than schizophrenia.

For patients with substance use disorders, numerous studies demonstrate impairments in social cognition. For example, studies with cannabis users were able to show alterations in the physiological response in tasks requiring empathy and ToM (9–11). ToM deficits have been found in cocaine users (12, 13) and methamphetamine users (14, 15). Studies researching patients with polysubstance abuse demonstrate correlations between ToM impairments and the number of consumed substances (16) as well as structural alterations in the orbitofrontal cortex (OFC) and anterior cingulate cortex (ACC). Both are structures associated with ToM and empathy (17, 18). Patients in opioid maintenance treatment (OMT) exhibit impairments in social perception such as reading facial expressions (19, 20), a construct linked closely to ToM (21). An exploratory study (22) comparing ToM data from a semi-structured interview and a movie paradigm in 29 opioid dependent patients and an equal number of healthy controls found that opioid dependent patients' performance was significantly lower in both ToM measures. However, there is still a lack of research exploring ToM deficits in opioid dependence and which factors might have an impact on ToM performance in opioid dependent patients.

### ToM and Opioid Dependence—Possible Influential Factors

#### Executive Functions

The relationship of ToM and executive functions has been studied in the field of developmental psychology as well as in regards to clinical populations. A recent meta-analysis (23) reported associations between ToM and executive functions in patients diagnosed with schizophrenia. Children with autism exhibit deficits in both ToM and executive functions, and those deficits appear to be associated (24, 25). Studies conducted with healthy preschool children demonstrate a link in the development of ToM and executive functions. Specifically, a strong link between inhibitory control and ToM was shown (26–28), with stronger correlations between ToM and conflict tasks, in which a dominant response had to be inhibited, compared to delay tasks, which required the delay of a response. Studies examining ToM in adults have found correlations between executive functions and age related changes in ToM performance in older adults, some also highlighting the relevance of inhibitory control (29, 30).

However, whilst the majority of publications suggest a link between executive functions and ToM, some studies show conflicting results. In a study by Cavallini et al. (31), executive functions did not predict age related ToM differences. Studies examining specific neurological conditions have found a disconnect between ToM and performance in executive functions in affected individuals [amygdala damage: case study by Fine et al. (32); frontal lobe lesions: Rowe et al. (33); multiple sclerosis: Batista et al. (34)]. Ahmed and Miller (35) offer an explanation for the varying results. In their study examining the relationship between ToM and executive functions using different ToM tasks, they suggest that different ToM tests are using different cognitive mechanisms, which may indicate that the ToM test used has an influence on the involvement of different components of executive functioning.

Research shows a link between opioid addiction executive function impairments (36, 37). These impairments appear to be not just a temporary effect of current drug use, but indeed a long term effect which is still detectable after several years of abstinence (38). Opioid agonists used in OMT have been linked to impaired executive functions (39). The duration of opioid maintenance therapy and the consumption of additional substances have previously been identified as the main factors influencing cognitive functioning, including executive functions, in opioid addicts (40). As deficits in executive functions have been linked to ToM impairments as well as opioid addictions, we hypothesized that ToM performance is associated with executive functions in opioid dependent patients.

#### Childhood Maltreatment

Childhood abuse and neglect are a potent source of stress and can lead to biochemical, structural and functional cerebral changes as a result of the stress response (41). Numerous studies have examined the effects of childhood trauma on social cognition. In a study by Burack et al. (42), children and adolescents who had experienced maltreatment exhibited delayed development of social perspective-taking abilities compared to non-maltreated peers. A link between childhood abuse and ToM impairments has been found in clinical and non-clinical samples. For example, women suffering from PTSD resulting from childhood abuse exhibit ToM deficits (43). In a study exploring ToM and childhood trauma in depressive patients and healthy controls, there was a correlation between ToM and emotional abuse in depressive patients and a correlation between ToM impairments and physical abuse in the control group. Childhood neglect however was positively associated with ToM in both groups (44). In a sample of chronically depressed patients, amygdala activation during affective ToM tasks was largely modulated by childhood maltreatment and not by pathophysiological correlates of depressive symptoms (45). In schizophrenic patients, ToM impairements have been associated with physical neglect during childhood (46) and childhood trauma has been related to alterations in brain functioning during a ToM task (47). In non-clinical groups, childhood maltreatment has been linked to ToM alterations in both children (48) and adults (49).

Adverse childhood events are a risk factor for substance dependence (50–52). Comparing nicotine-, cocaine-, and opioid dependent patients, the latter exhibit the highest prevalence of lifetime traumatic events (53). In a sample of 150 patients in methadone substitution treatment, 29% of patients met the diagnostic criteria for PTSD. Trauma-related symptoms were associated with more severe substance abuse (54). Regarding trauma and adversities occurring in childhood, there is a high prevalence of reported childhood maltreatment in opioid dependent patients (55). In a sample of 113 opioid dependent patients undergoing buprenorphine treatment, only 19.5 % reported not having endured any form of childhood trauma (56). Considering the high prevalence of childhood trauma in opioid dependent patients and the evidence indicating an association between childhood maltreatment and ToM, we hypothesized that ToM and experiences of childhood maltreatment are correlated in opioid dependent patient.

In this study, we aimed to gain a better understanding of ToM in opioid dependent patients undergoing opioid substitution treatment. Firstly, we wanted to see if the ToM deficits reported by Gandolphe et al. (22) could be replicated in our sample. Furthermore, we wanted to explore potential factors influencing ToM in opioid dependent patients. With this goal in mind, we tested patients' performance in executive functions tasks and collected data on childhood maltreatment, measures of psychological well-being and substance use related factors.

## Methods

### Participants

A sample of 66 opioid dependent patients undergoing opioid maintenance treatment (OMT) were recruited from three psychiatric outpatient centers in Mannheim, Germany. Study participation was voluntary and all participants provided written informed consent prior to participation. The study was approved by the ethics committee of the Medical Faculty Mannheim, Heidelberg University, Germany (AZ: 2018-531N-MA).

In order to compare ToM performance between opioid dependent patients and healthy individuals, a control group was generated from the data sample of healthy controls provided by Montag et al. (57) to match our sample of opioid dependent patients in age (+/– 3 years) and sex. For 17 out of 66 participants, it was not possible to find a match that met the criteria. For those participants, the closest respective match was selected from the sample. The resulting sample did not significantly differ from our sample of opioid dependent patients in age [t_(130)_ = 0.96, *p* > 0.05] or sex [χ^2^_(1)_ = 2.59, *p* > 0.05). The groups differed significantly in the level of education (see Table 1). However, the level of education was not associated with ToM performances in neither [opioid dependent patients: F_(3, 62)_ = 2.72, *p* > 0.05; healthy controls: F_(2, 55)_ = 2.82, *p* > 0.05]. Therefore, we considered this an acceptable difference between opioid dependent patients and control group. Demographic data are presented in Table 1.

**Table 1:** Demographic characteristics of study participants.

	**OMT patients (*n* = 66)**	**Healthy controls (*n* = 66)**	**Value**	***P***
**Sex**					
Male	68.2% (*n* = 45)	54.5% (*n* = 36)	*χ2*(1) = 2.59	0.08
Female	31.8% (*n* = 21)	45.5% (*n* = 30)	
Age	*M* = 43.38, *SD* = 8.62	*M* = 41.22, *SD* = 10.51	*t*(130) = 0.96	0.34
School degree	15.2% no degree	0% no degree	*χ2*(3) = 41.38	<0.001
	51.5% Hauptschule^a^	13.6% Hauptschule^a^		
	25.8% Realschule^b^	30.6% Realschule^b^		
	7.6% Abitur^c^	43.9% Abitur^c^		
Years of heroin abuse	*M* = 14.29, *SD* = 8.44			
Opioid substitution					
Methadone	34.8 % (*n* = 23)			
Polamidon	33.3 % (*n* = 22)			
Buprenorphine	30.3 % (*n* = 20)			
retarded morphine	1.5 % (*n* = 1)			
Take home prescription^d^	72.7 % no (*n* = 48)			
	27.3 % yes (*n* = 18)			
**Additional substance abuse**					
None	12.1% (*n* = 8)			
Heroin	15.2% (*n* = 10)			
Cocaine	9.1% (*n* = 6)			
Cannabis	34.8% (*n* = 23)			
Benzodiazepines	25.8% (*n* = 17)			
Alcohol	10.6% (*n* = 7)			
Pregabalin	10.6% (*n* = 7)			
Amphetamine	7.6% (*n* = 5)			
**Relevant psychiatric comorbidities**					
Borderline personality disorder	9.2 % (*n* = 14)			
Bipolar disorder	1.3 % (*n* = 2)			
Psychotic disorders	4 % (*n* = 6)			

a *German high school degree attained after 9 years;*

b *German high school degree attained after 10 years;*

c *German high school degree attained after 13 years;*

d *Patients with stable abstinence of any other substances than the prescribed OMT dose are eligible to visit the clinic only once per week and get a subscription for taking their daily dose at home. This is referred to as “Take Home”*.

### Procedure

During a first appointment, opioid dependent patients were screened for psychiatric diagnoses using the German translation of the Structured Clinical Interview for the DSM (58). The screening was conducted by trained clinical staff. Patients were interviewed about current substance use and history of addiction. Additionally, substance use was regularly evaluated by urine screenings in the outpatient centers. Patients filled in the trauma questionnaire during this first appointment.

The ToM task and neuropsychological assessments were conducted on a separate day to ensure that the appointment was not too long for patients to keep up their level of concentration. Patients were tested for alcohol intoxication before the testing using a breath test.

### Measures

#### Theory of Mind

ToM performance was tested using the Movie for the Assessment of Social Cognition [MASC: (59)]. The MASC consists of a 15 min long movie about four protagonists getting together for a dinner party. The video is paused 45 times and participants are presented with questions aiming at the protagonists mental states. The administration of the MASC took approximately 45 min per participant. The items cover various aspects of social cognition, including emotions of different valence, thoughts, intentions, first and second order false beliefs, irony and faux pas. Participants have to take verbal content and intonation, facial expressions and body language into account, which contributes largely to the ecological validity of the MASC. The questions are posed in a multiple choice format with four answer options. For each question, the answer options are categorized in the following way: “correct ToM” (correct mental state inferences), “no ToM” (the answer is not related to mental states), “exceeding ToM” (overmentalizing; over-interpretative mental state inferences) and “low ToM” (insufficient, overly simplified mental state inferences). Six additional questions that are unrelated to ToM are used as an attention control. Scores for “cognitive ToM” (inferences about thoughts and intentions, 27 items) and “emotional ToM” (inferences about emotions, 18 Items) were calculated in addition to the total score. Chronbach's α for the MASC is reported at 0.84 (59).

#### Executive Functions

A short neuropsychological tests battery for examining executive functions was administered on a computer. Before each task, the examiner explained the task to the participant and was available to clarify any remaining questions to ensure participants' understanding of the tasks. The administration of all tasks took ~15 min.

Delay Task: The five-trial adjusting delay task (60) is a short version of the delay discounting task. Participants were confronted with five choices between money available now and a larger amount of money available after a delay. Depending on the participant's choice on the first trial, the time delay on the following trials is adjusted up or down. The dependent measure for this task is the discount rate k, which indicates how much the value is affected by the delay. A higher discount rate indicates a higher depreciation of delayed rewards.

Dimensional Change Card Sort [DCCS, (61)]: This task was implemented to test participants' cognitive flexibility. Participants have to switch between sorting bivalent stimuli according to the criteria of shape and color using the left and right arrows on the keyboard. The resulting score is based on an algorithm taking both accuracy and reaction time into account. For detailed information on score calculation, see Zelazo et al. (61). The DCCS has a good test-retest reliability at ICC = 0.85 (61).

Stop Signal Task [SST, (62)]: This is a task for measuring response inhibition. Participants were instructed to push the right arrow key as fast as possible when a square appeared on the screen and the left arrow key when a circle appeared on the screen. However, when the shape appeared and changed color from blue to orange after 300 ms, they were instructed not to press any key and had to suppress the initial motoric response. Of the 150 randomized trials, 30 were stop-trials. As outcome variables we used the number of false alarms (pressing the key in a no go trial) and the stop signal reaction time (SSRT). The latter is a measure for the latency of the stop process and is estimated by subtracting the mean stop-signal delay from the median reaction time on go trials. A meta-analysis found the reliability of the SSRT measure to be good with an average ICC of 0.71 (63).

#### Childhood Trauma

Childhood maltreatment was assessed using the German translation of the Childhood Trauma Questionnaire (CTQ), a self-assessment instrument with five scales differentiating between physical and emotional abuse and neglect as well as sexual abuse. The internal consistency for the CTQ is reported at Chronbach's α = 0.94 (64).

### Statistical Analysis

Statistical analyses were carried out as indicated in the results section using IBM SPSS Statistics 25 for Windows. Results were regarded as significant when the two-sided *p*-value was below 0.05.

For the group comparison of ToM performance on the MASC, Mann-Whitney *U*-tests and multiple regression analyses were conducted. To explore associations between ToM performance, substance use related variables, executive functions and the CTQ, Pearson's correlations were administered. In this part of the analysis, only the data of the opioid dependent patients were analyzed, as the relevant data beyond MASC performance was not available for the control group.

## Results

### Group Characteristics

Opioid dependent patients and control group did not differ significantly with regards to sex [χ^2^_(1)_ = 2.59, *p* > 0.05] or age [t_(130)_ = 0.96, *p* > 0.10]. The level of education differed significantly between the two groups [χ^2^_(3)_ = 41.38, *p* < 0.001], with more participants of the control group having achieved higher educational degrees than of the opioid dependent group.

### ToM Performance

As MASC scores were not normally distributed in the control group, non-parametric tests were applied to analyze for group differences. To avoid alpha-inflation due to multiple testing, *p*-values were adjusted using the Bonferroni correction. Mann-Whitney *U*-tests revealed significantly lower MASC scores overall as well as for cognitive and emotional ToM scores and all three error scores (no ToM, exceeding ToM, low ToM) in the opioid dependent group (see Table 2). In a subanalysis opioid dependent patients with comorbid diagnoses that might affect ToM, that is, bipolar disorder, borderline personality disorder, and history of psychotic disorders were excluded, as well as patients who might be under the influence of substances. Here we used clinical intoxication at the time of testing or urine test positive for benzodiazepines or pregabalin on the testing day. In this subanalysis the difference in MASC scores remained statistically significant (*p* < 0.001).

**Table 2:** Descriptive statistics for MASC scores in opioid dependent patients and control group and group comparison with Mann-Whitney *U*-test.

**MASC variables**	**Opioid patients (*****n*** **=** **66)**		**Healthy controls (*****n*** **=** **66)**		**Mann-whitney** ***U*****-test**	
	**M**	**SD**	**M**	**SD**	***U*** **-value**	***P***
Total score	27.29	5.21	33.64	3.65	781.5	<0.001
Cognitive ToM	16.56	3.60	20.41	2.57	842	<0.001
Emotional ToM	11.23	2.49	13.23	1.56	1,185	<0.001
Exceeding ToM	7.05	3.05	4.59	2.36	1,315	<0.001
Low ToM	6.58	3.34	4.41	2.44	1322.5	<0.001
No ToM	3.59	2.63	2.02	1.32	1,401	<0.01

*P-values were adjusted using Bonferroni correction*.

A multiple linear regression analysis was conducted to predict MASC total scores based on group (opioid dependent patients or control group), sex, age, level of education and the number of correct control questions in the MASC using the enter method. A significant regression equation was found [F_(5, 115)_ = 19.917, *p* < 0.001]. The R^2^ for the overall model was 0.46 (adjusted R^2^ = 0.44), indicative for a high goodness-of-fit according to (65). Group (B = 2.51, *p* < 0.01), age (B = −0.12, *p* < 0.01), level of education (B = 1.34, *p* < 0.01) and the number of correct control questions (B = 1.36, *p* < 0.01) all contributed significantly to the model. Sex did not contribute significantly to the model (B = 1.3, *p* = 0.09).

A multiple linear regression using the forward selection method revealed that group (opioid dependent patients vs. healthy controls) was the predictor that could explain the most variance of MASC total scores on its own (adjusted R^2^ = 0.28). See Table 3 for model summary.

**Table 3:** Multiple linear regression predicting MASC total scores using the forward selection method.

**Model**	**Predictors**	**R^2^**	**Adjusted R^2^**	**Std. error of the estimate**	***F***	***P***	**β**	**Sig. of coefficients**
1	Group	0.28	0.28	4.59	47.32	<0.001	0.53	<0.001
2	Group	0.36	0.35	4.36	33.52	<0.001	0.40	<0.001
	Correct control questions						0.31	<0.001
3	Group	0.41	0.40	4.19	27.82	<0.001	0.37	<0.001
	Correct control questions						0.32	<0.001
	Age						−0.23	0.001
4	Group	0.45	0.43	4.08	23.8	<0.001	0.25	0.005
	Correct control questions						0.30	<0.001
	Age						−0.20	0.005
	Level of education						0.23	0.008

### ToM and Variables Related to Substance Abuse

There was no statistically significant difference in MASC total scores for the different substitution substances (methadone, polamidon, buprenorphine), F_(2, 62)_ = 2.02, *p* = 0.142. It did not have a significant effect on MASC scores if patients consumed additional psychoactive substances (*U* = 168, *p* = 0.54), had a Take Home prescription (*U* = 368.5, *p* = 0.36) or which substances they consumed. Based on the calculated Pearson's correlation coefficients, there were no significant correlations between MASC scores and number of consumed substances (r = 0.2, *p* = 0.15), the number of years of heroin use (r = −0.08, *p* = 0.55) or the number of years in OMT (r = −0.02, *p* = 0.89).

### ToM and Executive Functions

**Figure 1:**
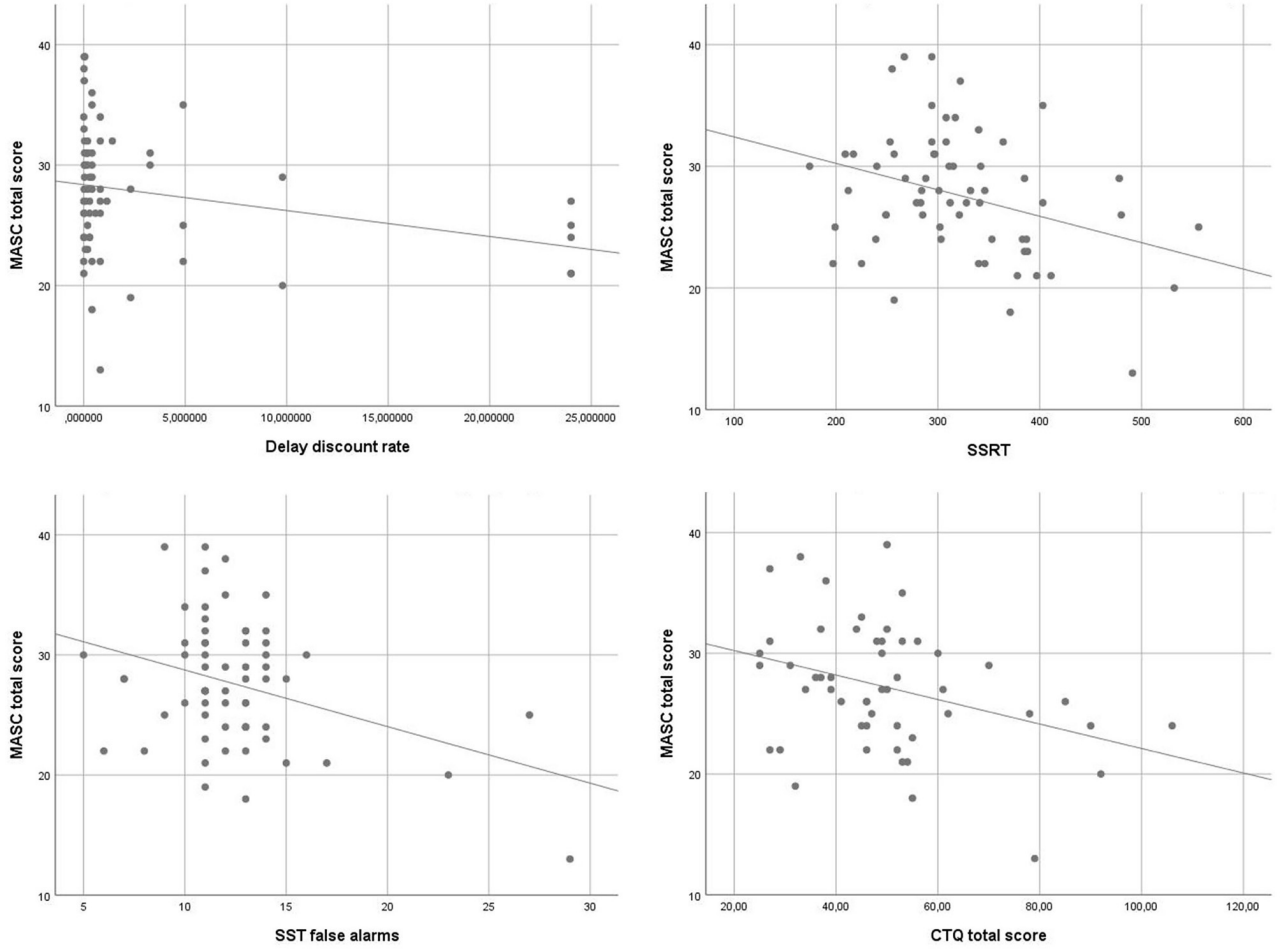
Scatterplots of correlations between MASC score, executive functions and CTQ.

To identify associations of ToM and executive functions, an exploratory analysis using Pearsons correlations was performed. MASC total scores correlated inversely with the delay discount rate (r = −0.27, *p* < 0.05), SSRT (r = −0.33, *p* < 0.01) and the number of false alarms in the SST (r = −0.35, *p* < 0.01). Furthermore, the emotional ToM score correlated inversely with SSRT (r = −0.40, *p* < 0.01) and the number of false alarms (r = −0.39, *p* < 0.01). The cognitive ToM score correlated inversely with the delay discount rate (r = −0.25, *p* < 0.05). Scatterplots of the main findings are depicted in Figure 1.

### ToM and Childhood Trauma

For the exploratory analysis of associations of ToM and childhood trauma also Pearsons correlations were performed. All *p*-values shown are corrected for multiple testing. The CTQ total score correlated inversely with the MASC total score (r = −0.34, *p* < 0.05) and the cognitive ToM score (r = −0.35, *p* < 0.05). The physical abuse scale correlated inversely with the MASC total score (r = −0.27, *p* < 0.05). The physical neglect scale correlated inversely with the MASC total score (r = −0.30, *p* < 0.05) and cognitive ToM (r = −0.30, *p* < 0.05). The remaining CTQ scales emotional abuse, emotional neglect and sexual abuse did not significantly correlate with the MASC.

## Discussion

The present study investigated ToM in opioid dependent patients undergoing OMT as well as possible influences on it. The first important result is the confirmation of poorer ToM performance in opioid dependent patients compared to healthy controls. This is in line with the findings of Gandolphe et al. (22). As the group difference was stable when controlling for the influence of other substance consumption and comorbid psychiatric diagnoses that might affect ToM, it seems like the found ToM deficits are associated with opioid dependence itself and not just due to the influence of intoxication or psychiatric comorbidities common in opioid users.

Substance use related variables like the substance used for OMT, duration of OMT, onset of opioid dependence, use and duration of use of non-opioidergic drugs, and eligibility for a Take Home prescription were not associated with ToM performance in the MASC in our sample. Again, this finding is reflected in the results of Gandolphe et al. (22), who found no link between patients' ToM abilities and substance use related variables (duration of substance abuse, age at onset of substance abuse and duration of abstinence). The authors interpret this as an indicator for ToM deficits predating substance abuse, highlighting ToM impairments as a suspected risk factor for substance abuse. For example, it might be possible that ToM deficits lead to more interpersonal problems and social stress resulting in increased substance use. As Gandolphe et al. (22) suggest, ToM deficits and their implication should be prioritized in the rehabilitation of opioid dependent patients.

The contribution of this study beyond the replication of previous findings is the exploration of possible influential factors on ToM in opioid dependent patients. Investigating the relationship between ToM and components of executive functioning, we found that MASC total scores correlated significantly with measures for delay discounting and response inhibition. The correlations were negative, indicating that opioid dependent patients who rejected delayed rewards at a higher rate and who had poorer inhibitory control exhibited weaker ToM. The DCCS score did not significantly correlate with any of the MASC scores, suggesting that set shifting does not play an important role in ToM performance. The SST variables correlated with the emotional ToM scale, whereas the delay discount rate correlated with the cognitive ToM scale. This result suggests that response inhibition plays a significant role in emotional ToM, but not in cognitive ToM. Furthermore, delay discounting appears to be associated with cognitive, but not emotional ToM. Different studies suggest a dissociation between cognitive and emotional ToM (66–68). Our findings seem to support the idea of different cognitive processes underlying cognitive and emotional theory of mind.

Regarding the relationship between ToM and childhood maltreatment, we found that the CTQ total score as well as both scales for physical maltreatment (physical abuse and physical neglect) were inversely correlated with the MASC total score. Higher scores on the CTQ and the aforementioned scales were associated with weaker ToM performance. This appears to be in line with the findings of Mrizak et al. (46), who reported a link between physical neglect in childhood and ToM deficits in schizophrenic patients. The role of physical childhood maltreatment in the ToM performance of opioid dependent patients suggested by this result appears noteworthy. It may be an indication that ToM impairments in opioid dependent patients suffering from childhood maltreatment are not due to emotional neglect, i.e., less opportunities for practicing certain aspects of social cognition, but rather suggests that influences of physical abuse and neglect (e.g., violent impacts to the head, malnourishment) on brain development may be at the core of the relationship between childhood trauma and ToM. Another explanation might be that physical and emotional childhood abuse and neglect often occur together. It is hard to imagine that a child who is abused on a physical level is simultaneously receiving the necessary emotional care and attention to the full extent. This assumption is backed by research: Adverse childhood events have been shown to be interrelated, with adults who reported one form of adverse childhood experience were likely to have experienced other forms as well (69). Claussen and Crittenden (70) found that most children experiencing physical maltreatment experienced psychological maltreatment as well and that the latter was a better predictor for negative outcomes than the severity of physical injury. Emotional abuse however is more likely to go under-identified by the person affected by it (71) as well as by other people (70). In this light, it can be assumed that patients who reported physical abuse and neglect in the CTQ might also have experienced forms of emotional abuse and neglect, but may not have reported it in the questionnaire to the full extent. Therefore, it can be argued that the correlation between physical maltreatment and ToM in our sample of opioid dependent patients may not be due to the isolated effects of physical maltreatment, but also might be influenced by concurrent emotional maltreatment.

As childhood trauma can influence the development of executive functions (72), it can be hypothesized that the correlations we found between executive functions and ToM in opioid dependent patients might be mediated by childhood maltreatment. A mediator analysis with a larger sample of opioid dependent patients investigating the relationship between ToM, executive functions and childhood maltreatment might help to bring further understanding of this matter. Furthermore, longitudinal studies examining the relationship between childhood maltreatment, executive functions, ToM and addiction would be an important step toward understanding how these factors play into each other.

In addition to executive functions, another relevant factor of cognitive functioning impacting ToM in opioid dependent patients could be intelligence. Correlations between measures of executive functioning and fluid intelligence have previously been reported (73). Studies have linked ToM performance to general intelligence (74), however, it is not fully clear how much of this association may be due to the language based nature of ToM tasks (75). Further research examining the relationship between ToM, executive functions and intelligence are needed to gain a deeper understanding of influences on impaired ToM in patients with substance use disorders.

Another perspective for future research could be the examination of the relationship between ToM and substance use related factors, executive functions and childhood trauma in other substance use disorders. Bosco et al. (76) found that the duration of alcohol abuse correlated negatively with ToM performance in alcohol dependent patients, suggesting brain damage due to the neurotoxic effects of alcohol to be a relevant factor for ToM impairment in this population. This differs notably from our finding, which might indicate different mechanisms underlying ToM impairment in opioid dependent patients and alcohol dependent individuals. Exploring and comparing influential factors on ToM in abusers of different substance groups in future research could be very beneficial to the understanding of the processes underlying social-cognitive impairments in substance use disorders.

The present study has some limitations that need to be mentioned. Firstly, the opioid dependence group and control group were not an identical match regarding age and sex. However, those differences were not of statistical significance. Furthermore, the groups did significantly differ in education. As we found no significant association between the level of education and ToM in either group, we concluded that the intended comparison of ToM between the groups would still be valid. Aside from that, the participants of the control group were tested in a different institution a number of years before the testing of our sample of opioid dependent patients. As there is no literature reporting cohort effects of ToM or social cognition in general, we considered this not to be a hindrance to the comparison of ToM performance between the groups. Also, the fact that the outcome in this study reflects what Gandolphe et al. (22) found in their sample points toward the validity of our group comparison. Therefore, we consider the group comparison of ToM between opioid dependent patients and healthy controls in this study to be cogent despite the described differences between the groups. Nonetheless, a comparison between ToM in opioid dependent patients and perfectly matched healthy controls in identical testing conditions would be desirable. Another potential limiting factor in this study is that a substantial percentage of the opioid dependent patients in our sample were also consuming substances other than opioids and/or had comorbid psychiatric diagnoses. One could therefore argue that group differences and correlations found in this study are not necessarily related to opioid addiction and opioid maintenance treatment per se, but might be related to other mental health and addiction variables. However, we did control for these factors in our group comparison analyses. Although it would have been possible to only recruit opioid dependent patients with no comorbidities and no additional substance use, these patients represent a minority of the population of opioid dependent patients. It was important to us to examine a representative sample of opioid dependent patients, which in our opinion enables more of a generalization of our findings to opioid dependent patients beyond our specific sample than it would be possible with the exclusion of comorbidities and other substance use.

In summary, the present study was able to confirm ToM deficits in opioid dependent patients in comparison with healthy controls and was the first to explore the influence of executive functions and childhood maltreatment on ToM in opioid dependent patients. And although there are certain limitations to this predominantly exploratory study, it provides a step toward a better understanding of ToM in opioid addiction. The dissociation of ToM and substance use related variables in combination with the correlations between ToM and childhood maltreatment strongly suggest that ToM impairments are not the mere consequence of opioid use, but may predate substance abuse and pose a risk factor for the development of opioid addiction. The protruding role of physical maltreatment might be a hint toward neurological factors playing a part in the development of ToM impairments in this population. Further research is needed to analyze the directionality of the relationships found in this cross-sectional study, as this could potentially be of interest from a clinical perspective, e.g., for the development of prevention programs targeting victims of physical childhood maltreatment or interventions aiming at the improvement of theory of mind which might contribute to the prevention of opioid addiction.

## Data Availability Statement

The datasets presented in this article are not readily available because participants did not consent to providing data to other institutions. Requests to access the datasets should be directed to Katharina Eidenmueller, katharina.eidenmueller@zi-mannheim.de.

## Ethics Statement

The studies involving human participants were reviewed and approved by Ethics Committee of the Medical Faculty Mannheim, Heidelberg University, Germany. The patients/participants provided their written informed consent to participate in this study.

## Author Contributions

Material preparation and data collection were performed by KE, FG, and NB. Data analysis was performed by KE and supervised by DH. The first draft of the manuscript was written by KE. All authors contributed to the study conception and design, worked on reviewing and editing of the manuscript, read and approved the final manuscript.

## Conflict of Interest

The authors declare that the research was conducted in the absence of any commercial or financial relationships that could be construed as a potential conflict of interest.

## Publisher's Note

All claims expressed in this article are solely those of the authors and do not necessarily represent those of their affiliated organizations, or those of the publisher, the editors and the reviewers. Any product that may be evaluated in this article, or claim that may be made by its manufacturer, is not guaranteed or endorsed by the publisher.
